# Excellent outcomes among HIV+ children on ART, but unacceptably high pre-ART mortality and losses to follow-up: a cohort study from Cambodia

**DOI:** 10.1186/1471-2431-9-54

**Published:** 2009-08-20

**Authors:** Marie-Eve Raguenaud, Petros Isaakidis, Rony Zachariah, Vantha Te, Seithabot Soeung, Kazumi Akao, Varun Kumar

**Affiliations:** 1 Médecins Sans Frontières, 72, Street 592, Phnom Penh, Cambodia; 2 Médecins Sans Frontières, Operational Research Unit, 94 rue Dupre, 1090 Brussels, Belgium; 3 Pediatric Department, Donkeo Referral Hospital, Takeo, Cambodia; 4 Angkor Hospital for Children, Siem Reap, Cambodia

## Abstract

**Background:**

Although HIV program evaluations focusing on mortality on ART provide important evidence on treatment effectiveness, they do not asses overall HIV program performance because they exclude patients who are eligible but not started on ART for whatever reason. The objective of this study was to measure mortality that occurs both pre-ART and during ART among HIV-positive children enrolled in two HIV-programs in Cambodia.

**Methods:**

Retrospective cohort study on 1168 HIV-positive children <15 years old registered in two HIV-programs over a four-year period. Mortality rates were calculated for both children on treatment and children not started on ART.

**Results:**

Over half (53%) of children were 5 years or above and only 69(6%) were <18 months. Overall, 9% (105/1168) of children died since the set-up of the programs. By the end of the observation period, 66(14.5%) patients not on ART had died compared to 39(5.5%) of those under treatment, and 100(22%) who did not start ART were lost-to-follow-up compared to13(2%) on ART. 66/105 (62.8%) of all in-program deaths occurred before starting ART, of which 56% (37/66) and 79% (52/66) occurred within 3 and 6 months of enrolment respectively. Mortality rate ratio between children not on ART and children on ART was 4.1 (95%CI: 2.7–6.2) (P < 0.001). The most common contributing cause of death in first 3 months of treatment and in first 3 months of program enrollment was tuberculosis. 41/52 (79%) children who died within 6 months of enrollment had met the ART eligibility criteria before death.

**Conclusion:**

HIV-positive children experienced a high mortality and loss-to-follow-up rates before starting ART. These program outcomes may be improved by a more timely ART initiation. Measuring overall in-program mortality as opposed to only mortality on ART is recommended in order to more accurately evaluate pediatric HIV-programs performance.

## Background

Anti-retroviral treatment (ART) significantly decreases mortality and pediatric HIV care programs in resource-limited settings achieve treatment outcomes similar to those in industrialized countries [1-5]. The success of pediatric HIV/AIDS care programs has been generally assessed by measuring the response of children placed on ART. Although such program evaluations provide important evidence on treatment effectiveness, they do not correctly assess overall HIV program performance as the cohort that is assessed excludes individuals who are eligible but not started on ART for whatever reason.

Thus the current "snapshot" is restrictive and little is known about deaths occurring prior to ART initiation (pre-ART mortality), which go unreported.

Information on such "hidden mortality" from programmatic settings could bring a better understanding on this issue and help identify ways to optimize monitoring and assessments of overall performance of pediatric HIV/AIDS programs.

In the past five years the Ministry of Health, Médecins Sans Frontières (MSF), and Angkor Hospital for Children (AHC) have been supporting pediatric HIV/AIDS care programs in Takeo and Siem Reap provinces in Cambodia. Overall, excellent clinical, immunological and virological outcomes under ART were achieved in these two pediatric cohorts [2].

We conducted a retrospective analysis of program data in order to measure mortality occurring both before (pre-ART) and during ART among HIV-positive children enrolled for HIV/AIDS care in two hospitals in Cambodia.

## Methods

### Study setting and population

The study was conducted in the pediatric HIV clinics of the Angkor Hospital for Children (AHC) a charity hospital located in Siem Reap province (population 700 000), and in Donkeo Referral Hospital, a public facility in Takeo province (population 800 000). The majority of the population live in rural areas and their socioeconomic status is very low. All children <15 years of age with confirmed HIV-positive status registered in these two HIV programs from January 2003 until December 31 2007 were included in the study. Children switched at any time to second line therapy were also included in the analysis.

The HIV clinics are located within the hospital and operate on an out-patient basis, providing doctor-based clinical care and follow-up by a multi-disciplinary team including counselors, drug-educators and nurses. Most patients are self-referred or referred by private or public health facilities in the province. In both study locations a prevention of mother to child transmission (PMTCT) program was in place albeit with limited coverage. On average, one fifth of children enrolled in the program in Takeo entered through the PMTCT program (no data available for Siem Reap location). Virological testing (detection of plasma HIV-1 RNA by RT PCR) for HIV diagnosis in children younger than 18 months of age was made available during the year 2006. Prior to that time, children were identified as having presumptive HIV infection on the basis of clinical signs and symptoms, with diagnosis confirmed by antibody testing at 18 months.

The patient assessment at first consultation included a history and physical examination as well as determination of clinical disease stage using CDC clinical categories [6] (from 2003–early 2006) and WHO criteria (2006–2007) [7,8]. If the patient was in an advanced stage of disease (WHO 3 or 4, CDC "C"), s/he was followed up on a monthly basis or more frequently if this was merited by clinical status. If the patient was in WHO stage 1 or 2 or CDC stage A or B, appointments were given on two monthly basis. Blood sampling was generally performed on admission (or upon confirmation of HIV status) for CD4 count or percentage measurement and every 6 months thereafter. Average delay in obtaining CD4 results was one to three weeks. In the early years of the program the protocol recommended to repeat CD4 a second time after 1–3 months in order to confirm a low CD4 value before starting ART.

At each visit a standardized admission or follow up sheet was filled in by the doctor.

An active tracing system for patients who failed to attend a clinic appointment was set-up, and included home visits and additional counseling sessions. Due to limited resources, such defaulter tracing system was reserved mainly for children who were initiated on ART.

### ART eligibility, regimens and treatment outcomes

Criteria to start ART in HIV-positive children were: WHO stage 3/4 or CDC stage C or CD4 < 200 cells/mm^3 ^(for individual ≥ 5 years of age) or CD4% < 20.0% in Siem Reap (for individuals < 5 years of age) or CD4% < 15.0% in Takeo (for < 5 years of age). As of 2006 protocols were standardized for both locations and the new WHO criteria were used (CD4 < 200 cells/mm^3 ^for children ≥ 5 years, CD4% < 15% for children 36–59 months, and CD4% < 20% for children 12–35 months) [8]. According to the same clinical and immunological criteria, children were offered fluconazole and cotrimoxazole prophylaxis, provided there were no contraindications.

In one clinic at the beginning of the program a maximum of five children per month were started on ART due to limited capacity for follow-up, creating a backlog of patients.

The majority of patients were started on a standard first-line regimen of stavudine, lamivudine and nevirapine. Zidovudine and efavirenz were used as alternatives in case of intolerance or interaction with other drugs. Patients and caregivers received at least three ART-preparation counseling sessions, one week apart, before starting treatment. Potential reasons for deferral of treatment included active opportunistic infection(s), lack of caregiver readiness or a failure to attend consecutive follow-up appointments indicating potentially problematic adherence to treatment.

Outcomes were standardized and included: alive and on active follow-up, died, transferred out, and lost-to-follow-up (>3 months) (LTFU). Most transfers took place after 2005 as new pediatric HIV clinics started operating in the country. All medical services were offered free of charge for HIV-positive children and additional support was provided by various Non-Governmental Organizations (NGOs) including transportation, accommodation and food allowance.

### Data management and statistical analysis

Information from structured patient files, admission and follow up sheets, were entered routinely by data clerks into a HIV-specific database (FUCHIA 1.6.0 developed by EPICENTRE, Paris, France. (available at ). Data entry was cross-checked and updated from patient files.

To accommodate for the fact that immunological criteria for severe HIV immunodeficiency were age-specific, we created a categorical variable describing the presence of severe immunodeficiency according to the WHO 2006 criteria (18–35 months: <20% CD4% or <750 CD4 count; 36–59 months: <15% CD4% or <350 CD4 count; ≥ 59 months: <15% CD4% or <200 CD4 count). As both the CDC clinical staging system and the WHO staging system were used by the programs, WHO stages 3 and 4 were grouped together with CDC grade C as an indicator of advanced AIDS disease. Date of ART eligibility was calculated for each patient using the CD4-based ART initiation criteria and information from the patient file. The most likely attributable cause of death of each patient was assigned based on the last diagnosis available in patient records.

Person-time analysis was used for the mortality rate. The observation period went up to April 2008 when the data set was censored. We provided the outcomes at 1 year after enrollment. Data analysis was performed using STATA 8.2 software (Stata Corporation, College Station, Texas, USA).

As this was a retrospective study using program data, informed consent from individual patients was not obtained. The study protocol was approved by the Ethics Review Board of Médecins Sans Frontières and by the Institutional Review Board of Angkor Hospital for Children.

## Results

### Enrolment, follow up and main outcomes

1168 HIV-positive children were registered in the two HIV clinics between January 2003 and December 2007, and included in the analysis. Over half (53%) of children were 5 years or above and only 69 (6%) were less than 18 months. 148 (27%) children ≥ 5 years had first CD4 lymphocyte count < 50 cell/μl (data available for 87% of children). 164 (41%) children aged 18–59 months had first CD4 lymphocyte percentage < 15% (data available for 88% of children). The characteristics of patients at time of registration are shown in Table 1.

**Table 1:** Characteristics of children enrolled in the HIV programs at the time of registration and their outcomes.

	<18 months	18–59 months	≥ 60 months	Total
Number of children registered	69 (5.9%)	479 (41.0%)	620 (53.1%)	1168

Gender				
Female	33 (47.8%)	230 (48.0%)	292 (47.1%)	555 (47.5%)

WHO or CDC stage				
WHO 1/2 or CDC stage N/A/B	34 (50.0%)	310 (64.7%)	395 (63.7%)	739 (63.3%)
WHO 3/4 or CDC C	34 (50.0%) (n = 68)	169 (35.3%)	225 (36.3%)	428 (36.7%) (n = 1167)

CD4 cells/mm^3^, median (IQR)	753 (338–1280) (n = 40)	643 (310–1049) (n = 353)	245 (40–599) (n = 561)	410 (94–818) (n = 954)

CD4 cells/mm^3^				
<50	2 (5.0%)	22 (6.2%)	149 (26.6%)	173 (18.1%)
50–350	8 (20.0%)	75 (21.3%)	175 (31.2%)	258 (27.0%)
>350	30 (75.0%)	256 (72.5%)	237 (42.2%)	523 (54.8%)

CD4%, median (IQR)	18.71(12.44–23.75) (n = 48)	18 (10–24.8) (n = 415)	9.41 (2.76–18.72) (n = 399)	14.47 (5.33–23) (n = 862)

CD4% group^3^				
<15.0%	16 (33.3%)	164 (39.5%)	260 (65.2%)	440 (51.0%)
15–24.9%	21 (43.7%)	148 (35.7%)	81 (20.3%)	250 (29.0%)
≥ 25.0%	11 (22.9%)	103 (24.8%)	58 (14.5%)	172 (20.0%)

Weight for height*				
≥ -2 z-score	33 (55.0%)	366 (81.0%)	435 (76.7%)	834 (77.3%)
<-2 and ≥ -3 z-score	12 (20.0%)	53 (11.7%)	82 (14.5%)	147 (13.6%)
<-3 z-score	15 (25.0%)	33 (7.3%)	50 (8.8%)	98 (9.1%)

Weight for age				
≥ -2 z-score	29 (42%)	153 (31.9%)	127 (20.5%)	309 (26.5%)
<-2 and ≥ -3 z-score	12 (17.4%)	176 (36.7%)	287 (46.4%)	475 (40.7%)
<-3 z-score	28 (40.6%)	150 (31%)	205 (33.1%)	383 (32.8%)

Year of admission				
2003	2 (2.9%)	53 (11.1%)	36 (5.8%)	91 (7.8%)
2004	13 (18.8%)	117 (24.4%)	183 (29.5%)	313 (26.8%)
2005	17 (24.6%)	123 (25.7%)	165 (26.6%)	305 (26.1%)
2006	19 (27.5%)	109 (22.8%)	130 (21.0%)	258 (22.1%)
2007	18 (26.1%)	77 (16.1%)	106 (17.1%)	201 (17.2%)

Started ART§	42 (60.9%)	278 (58.0%)	394 (63.6%)	714 (61.1%)

Follow up time in days, median (IQR)	333 (134–840)	755 (349–1207)	750.5 (320.5–1193)	733.5 (314.5–1182.5)

Outcome on study termination:				
Alive and in active follow up	40 (58.0%)	361 (75.4%)	448 (72.3%)	849 (72.7%)
Dead	15 (21.7%)	37 (7.7%)	53 (8.6%)	105 (9.0%)
Lost to follow up*	8 (11.6%)	42 (8.8%)	63 (10.2%)	113 (9.7%)
transferred 6 (8.7%)	39 (8.1%)	56 (9.0%)	101 (8.6%)	

WHO: World Health Organization; CDC: Centre for Disease Control; IQR: inter-quartile range; ART: anti-retroviral treatment

last contact with clinic > 3 months

§32 patients switched to second line therapy during the study period

CD4 count and %: measured within 120 days after date of registration or HIV status confirmation

During the study period, 714 (61%) children started ART. The median period of observation for patients not receiving ART was 11 months (IQR 2.4–25). Among the 454 patients who did not start ART, 66 (14.5%) had died, 100 (22%) were lost-to-follow-up, 40(9%) were transferred out, and 248 (55%) were still enrolled in the program and on active follow-up.

For children who started ART, the median interval between registration and ART initiation was 4.7 months (IQR 2.5–8.8). The median period of observation under treatment was 2 years (IQR 0.9–3.0). By the end of the observation period 39 (5.5%) patients under treatment had died, 13 (2%) were lost-to-follow-up, 61 (8.5%) were transferred to other programs, and 601 (84.2%) were still alive and on follow up.

**Figure 1:**
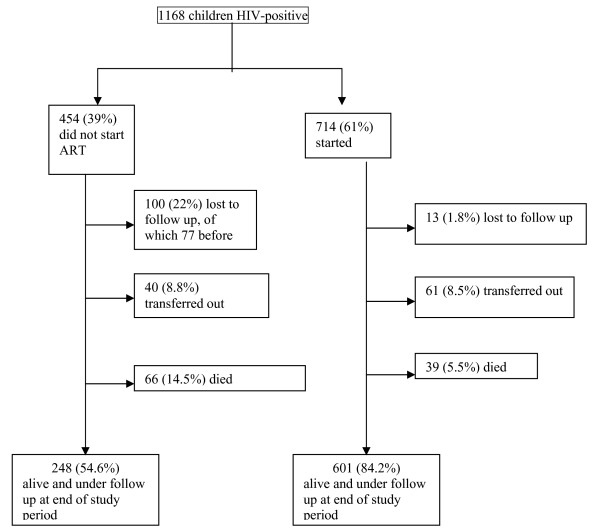
**Cohort outcomes of children who did and did not start ART after enrollment in HIV programs**. Very early deaths – deaths within 3 months of enrollment.

**Figure 2:**
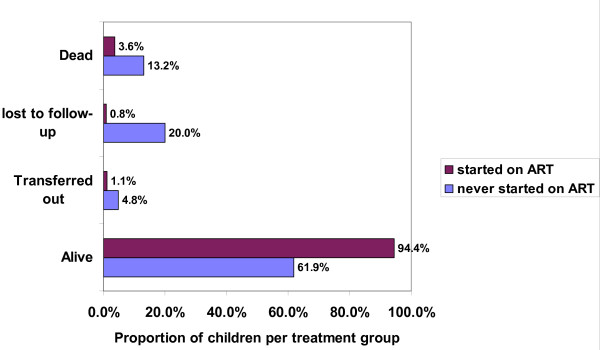
**Outcomes of HIV-positive children 1 year after enrolment in program**. ART: anti-retroviral therapy.

The main outcomes of children who started ART and those who never started ART after 1 year of enrollment in the HIV care program are shown in Figure 1 and Figure 2. 27/248 (11%) of children alive at the end of the observation period were actually eligible for treatment.

### Mortality

Overall, 9% (105/1168) of children died from the beginning of the program. Overall in-program mortality rate was 4.27 deaths per100 person-year (95%CI: 3.5–5.2). 66/105 (62.8%) of all in-program deaths occurred before starting ART, of which 56% (37/66) occurred within 3 months of enrollment, and 23% (15/66) between 3 and 6 months after enrollment. Table 2 shows the mortality rates for children who received treatment by measuring follow up time from start of ART in the first 3 months, from 3–6 months and from 6 months onwards, and compares these with the rates in children who never received ART measuring follow up time from enrolment.

**Table 2:** Mortality in HIV-positive children (on ART and never on ART) enrolled in the HIV programs.

	Person-time **on ART **(from ART initiation)			Person-time **never on ART **(from admission)		
Time period	Deaths	Person-years	Rate per 100 PY (95%CI)	Deaths	Person-years	Rate per 100 PY (95%CI)

< 90 days	20	168	11.9 (7.7–18.4)	37	92	40.0 (29.0–55.2)

90–180 days	7	322	2.2 (1.0–4.5)	15	170	8.8 (5.3–14.7)

> 180 days	12	1443	0.8 (0.5–1.5)	14	596	2.3 (1.4–4.0)

Overall mortality rate was four times higher among children never started on ART than among children started on ART (incidence rate ratio = 11.1/2.7 = 4.1; 95%CI:2.7–6.2; P < 0.001).

### ART eligibility of patients with early death

In order to investigate whether the early deaths among patients not treated could have been prevented by earlier treatment, we looked at whether patients had met the ART eligibility criteria before death and the time between eligibility and death. Out of 52 children not on ART who died within 6 months after enrolment, 41 (79%) had fulfilled the ART eligibility criteria at some point of time. Fourteen (27%) of them died within 0–30 days after eligibility criteria were met, 13 (25%) between 31–60 days, 5 (10%) between 61–90 days, and 9 (17%) after 90 days.

Fourteen children (13% of all deaths) died within the first month after admission and prior to ART initiation. Median age was 49 months (IQR: 27–78).

### Children lost to follow-up prior to ART initiation

Among 454 children who never started ART, 100 (22%) children were lost-to-follow-up within the first six months after registration, of whom 26 only came for one consultation. As there was no active tracing of these patients, eventual deaths occurring at home could not be ascertained. Baseline characteristics of the children lost-to-follow-up were: median CD4 count 402 (IQR: 22–664) and CD4% 18.3 (IQR: 4.1–23.6) (for children ≥ 60 and <60 months respectively), 45 had W/A z-score <-3, 73 lived outside the province, 93 were aged 18 months or above, and 72 were registered before 2006.

### Cause of early death

Contributing causes of very early death were assigned for all children not started on ART and for 16/20 children on ART. Among children not receiving ART, the most common illnesses contributing to death were tuberculosis (15 children, 41%), lower respiratory tract infections (9 children, 24%), diarrhea (4, 11%) and wasting syndrome/severe malnutrition (4 children, 11%). Among children on ART, tuberculosis was the most common illness (7 children, 44%) and respiratory tract infections (4 children, 25%).

## Discussion

In this study looking at in-program mortality in a large cohort of HIV-positive children enrolled in hospital-based HIV programs in two provinces of Cambodia, we found that although on-ART mortality was low, the pre-treatment mortality and losses to follow-up were unacceptably high. More than 6 out of every 10 deaths and almost 9 out of every 10 children lost to follow up occurring after enrollment in the HIV/AIDS programs happened among children not yet started on ART. Loss to follow up was significantly higher among non-treated children than among children on antiretroviral treatment. We suspect that many of the children lost-to-follow-up eventually died and it is likely that all these deaths were not accounted for in this group of patients. Mortality was, therefore, possibly underestimated due to misclassification of unascertained patient deaths as lost-to-follow-up.

Interestingly, among patients not started on ART, we found that there were 73/100 cases LTFU who lived outside the province and 161/354 (45.5%) cases not LTFU who lived outside the province (OR 3.2; 95%CI: 1.96–5.36); P < 0.001). Age and sex were not associated with LTFU (data not shown). This suggests that longer travelling distances made access to care more difficult to the point that children stopped coming back for consultation.

Despite the higher mortality rate for children not started on ART recorded in our program, the mortality rate was much lower than that for HIV-infected children measured among Zambian children prior to availability of ART [9]. Such large difference may reflect the positive impact of non-ART HIV care like cotrimoxazole prophylaxis, which was universally implemented in our setting, and aggressive treatment of opportunistic infections [10].

Mortality among children on treatment in our program was low and comparable to other settings of high HIV prevalence [1]. Moreover, the very low dropout rate (2%) among children on ART suggested they were better followed-up than those not on ART. This was a likely consequence of the "active" tracing system, which was unfortunately reserved for children on ART because these activities, including home visits and social support, were resource-demanding and beyond the program capacity. There are various factors that can explain the relatively low on-treatment mortality observed in our setting, even though a third of children were severely immunosuppressed on admission. The low number of infants in the cohort suggests that our in-program mortality reflects the mortality among children who survived the first year of life. Yet, it is known that most HIV deaths in children occur during this crucial time [4,5]. In our program the death ratio was highest in the youngest age group (<18 months). This could be partly explained by the fact that in the absence of good coverage of early diagnosis, symptomatic infants were more likely to be identified in the program than non-symptomatic ones. Although we could not retrieve individual patient information on cotrimoxazole and fluconazole prophylaxis, the systematic use of prophylaxis was recommended throughout the program, and would have contributed positively to limiting the mortality. Deaths occurring within one month of admission and, to a certain extent, the early deaths occurring within three months of admission are likely to reflect the late arrival of children with advanced HIV/AIDS disease.

TB prevalence is high in Cambodia at 665 per 100 000 persons per year [11] and it is the most common opportunistic infection among HIV patients in the country. In this study, TB was the most common cause of death, in nearly half of patients whether on ART or not. The challenges of diagnosing TB among HIV-positive children may have contributed to this problem. Prevention, screening, and treatment need to be optimized in order to reduce TB related mortality. Moreover, the lack of clear recommendations on the optimal time for starting ART during TB treatment in children and the fear of paradoxical reactions have likely contributed to clinicians delaying ART initiation in children with active TB. The decision to start ART during TB treatment is essentially a clinical judgment by the physician [12].

Most of the children who died before starting treatment had met the ART eligibility criteria (41/46), and could thus have started ART before the time of death. Overall, the average time to initiate ART (median of 4.7 months) in the program was relatively long, although comparable to community-based HIV programs in South Africa [13]. Some reasons for the delay in starting ART in our setting included lack of experience of clinicians in the early years of the program and the unnecessary requirement for two CD4 values below threshold before ART initiation. Additionally, the time intervals between CD4 measurements were long and there was no systematic recall of children with low CD4 counts as children only came on their scheduled appointment. Finally, delays in the identification and training of a caregiver, especially if child was orphan, were observed.

**Figure 3:**
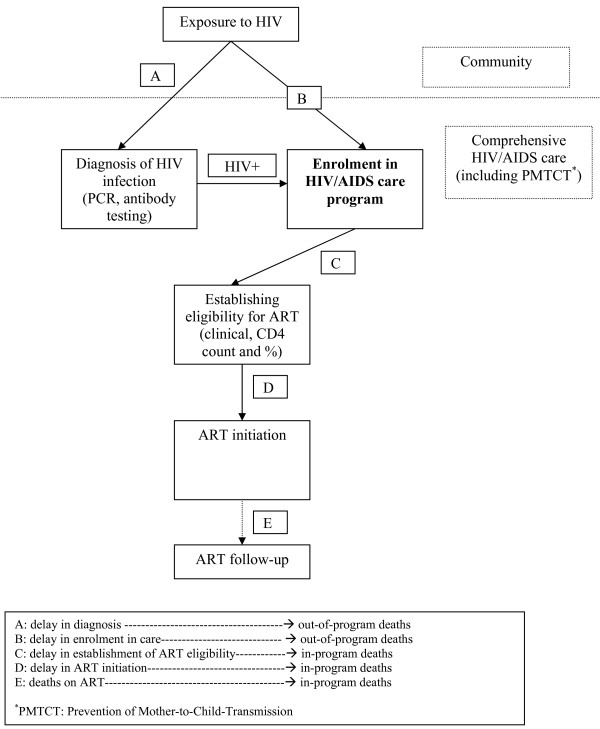
**Delays and deaths in pediatric HIV/AIDS care**.

From our experience we were able to identify three categories of reasons for delays in treatment initiation: first, delays in confirming HIV infection and enrolment in the HIV/AIDS care program for HIV-exposed and infected children (A & B in Figure 3); second, delays in establishing the degree of HIV immunodeficiency using CD4 measurements (C in Figure 3); and third, delays in initiating ART after ART eligibility criteria (clinical and/or immunological) were met (D).

Measures that contribute to minimizing these delays are urgently needed to help reduce the pre-treatment mortality and, possibly, the high loss-to-follow-up rates. Measures to speed up admission to the HIV clinic include early diagnosis and an efficient referral process. This implies the scale-up of HIV testing among infants and young children, in all out-patient services and in hospital wards, systematic HIV screening of children diagnosed with TB and overall an efficient prevention of mother-to-child-transmission program. Systematic DNA PCR testing in newborns is becoming increasingly available and would promote early HIV diagnosis and referrals for ART.

Delays in initiating ART after enrolment to the program could be minimized by the following:

1. Preparatory ART sessions could be scheduled as soon as the diagnosis of HIV infection is made and before ART eligibility criteria are met.

2. Access to CD4 measurements needs to be improved and CD4 monitoring optimized to facilitate timely initiation of ART: more frequent serial CD4 counts, and rapid feedback of results from laboratory. Should there be delays in CD4 measurements, clinical criteria for presumptive diagnosis of severe HIV disease should be used to initiate ART [8], while for infants, ART should be initiated regardless CD4 count [14,15].

3. A fast-track channel for children first admitted with advanced disease should be set-up: as soon ART eligibility is established, prompt recall of patient should be made without waiting for their next scheduled appointment.

4. If resources are available, a tracing system that is inclusive of all children (not just those on ART) missing a clinic appointment could help reduce loss-to-follow-up prior to ART initiation.

Strengths of this study include the large number of patients and the fact that data came from a program setting, thereby reflecting the operational reality on the ground.

However, there are certain limitations to the study. First, not all causes of death were reliably ascertained. Nevertheless, the level of error in attributing the cause of death was limited, as both HIV-programs were situated within a provincial referral hospital and medical doctors performed all consultations. Second, the not-treated patient group had a higher lost to follow-up rate than the on-treatment patient group (22% versus 1.8%) so there were likely more unreported deaths in the not-treated group. Consequently, the mortality rate ratio between the two groups would be underestimated.

The relatively low mortality observed among children on ART in these two pediatric treatment programs suggests that these programs are providing effective care. However, high in-program deaths and losses-to-follow-up prior to ART initiation highlights the fact that relying solely on ART outcomes as an indictor to assess program effectiveness provides a distorted view of the overall performance of pediatric HIV-care. Finally, we were not able to systematically document reasons for delaying treatment. Our findings warrant a separate investigation on specific reasons for ART delay including multivariate analysis of parameters associated with non-treatment.

## Conclusion

This study has shown that the measurement of in-program mortality helps reveal obstacles in the provision of care to HIV-positive children. It highlights challenges that remain to be tackled in order to further optimize the care for these children. Further studies should be done to look at effective ways of reducing the delay to ART initiation after meeting ART criteria and also whether less stringent criteria for starting ART would lead to better outcomes.

## Competing interests

The authors declare that they have no competing interests.

## Authors' contributions

MER designed the study, performed the statistical analysis and drafted the manuscript. PI participated in the study design, implementation of the program, and contributed to the draft manuscript. RZ participated in the study design and contributed to the draft manuscript. KA, VK, and SS participated in the study design and data analysis. All authors contributed to the data analysis, read and approved the final manuscript.

## Pre-publication history

The pre-publication history for this paper can be accessed here:


